# P-2250. Metagenomic Next Generation Sequencing Enhances Rickettsial Infection Diagnosis in Patients with Acute Undifferentiated Fever

**DOI:** 10.1093/ofid/ofae631.2403

**Published:** 2025-01-29

**Authors:** Sonja Weiss, Pakpoom Phoompoung, Julie Yamaguchi, Michael G Berg, Francisco Averhoff, Gavin Cloherty, Yupin Suputtamongkol

**Affiliations:** Abbott Labs, Abbott Park, Illinois; Siriraj Hospital / Mahidol University, Thailand, Bangkok, Krung Thep, Thailand; Abbott Labs, Abbott Park, Illinois; Abbott Labs, Abbott Park, Illinois; Abbott Laboratories, Atlanta, Georgia; Abbott, Abbott Park, Illinois; Mahidol University, Bangkok, Buriram, Thailand

## Abstract

**Background:**

Murine and scrub typhus are common causes of acute undifferentiated fever (AUF) in tropical regions, after malaria, dengue fever, and typhoid fever have been ruled out. While indirect immunofluorescence assay (IFA) can diagnose both diseases, accuracy depends on when the sample is collected. To improve detection, we used unbiased metagenomic Next Generation Sequencing (mNGS) on 969 AUF patient samples with negative IFA results from Siriraj Hospital, Bangkok, Thailand collected between 2015 and 2021.

**Methods:**

Plasma was pre-treated with benzonase before extraction on an Abbott *m*2000 instrument. mNGS libraries were prepared from double-stranded cDNA with Illumina Nextera XT reagents then sequenced on a NextSeq. Reads were taxonomically classified by Abbott pipelines SURPI or DiVir and mapped in CLC Bio Genomics Workbench (Qiagen, Inc).

**Results:**

During the study period, a total of 2,465 samples were analyzed by IFA, identifying 58 (2.4%) cases of murine typhus and 26 (1.2%) cases of scrub typhus. Among IFA negative samples, mNGS identified *Rickettsia* spp. in 27 cases (2.8%), including *R. typhi* (murine typhus) (n=19), *R. felis* (n=5), and *R. rickettsii*, *R. prowazaki*, and *R. australis* (n=1 each). Notably, mNGS identified *Orientia tsutsugamushi* (scrub typhus) in one sample missed by IFA.

For patients with mNGS-detected *R. typhi*, serological confirmation was achieved in 2 cases. Another 13 were classified as probable based on clinical presentation, while 4 cases lacked conclusive clinical correlation. For patients with *R. felis* identified by mNGS, 4/5 had confirmed or probable diagnoses based on clinical symptoms. The inconclusive case was a 65-year-old man with a urinary tract infection who died from sepsis for which mNGS revealed a co-infection with *R. felis* and *Serratia marcescens*. Interestingly, none of the mNGS positives for *R. r*ickettsii, *R. australis*, and *R. prowazaki* had clinical presentations that supported the diagnoses.

**Conclusion:**

mNGS significantly enhances the diagnosis of rickettsial infections, particularly for murine typhus. This improved diagnostic capability provided by mNGS holds promise for improved management of AUF patients, potentially reducing unnecessary testing and inappropriate antibiotic use.

**Disclosures:**

Sonja Weiss, BS, Abbott Laboratories: Employee Julie Yamaguchi, BS, Abbott Laboratories: Employee Michael G. Berg, PhD, Abbott Laboratories: employee|Abbott Laboratories: Stocks/Bonds (Public Company) Francisco Averhoff, MD, MPH, Abbott Laboratories: Employee Gavin Cloherty, PhD, Abbott Laboratories: Employee

